# Infancy predictors of Functional Somatic Symptoms in pre- and late adolescence: a longitudinal cohort study

**DOI:** 10.1007/s00431-024-05850-7

**Published:** 2024-12-02

**Authors:** Lina Münker, Martin Køster Rimvall, Lisbeth Frostholm, Eva Ørnbøl, Kaare Bro Wellnitz, Pia Jeppesen, Judith Gerarda Maria Rosmalen, Charlotte Ulrikka Rask

**Affiliations:** 1 Department of Child and Adolescent Psychiatry, Aarhus University Hospital Psychiatry, Palle Juul-Jensens Boulevard 175, 8200 Aarhus N, Denmark; 2 Department of Functional Disorders and Psychosomatics, Aarhus University Hospital, Aarhus, Denmark; 3 Department of Clinical Medicine, Aarhus University, Aarhus, Denmark; 4 Department of Child and Adolescent Psychiatry, Copenhagen University Hospital - Psychiatry Region Zealand, Roskilde, Denmark; 5 Child and Adolescent Mental Health Centre, Copenhagen University Hospital - Mental Health Services CPH, Copenhagen, Denmark; 6 Department of Clinical Medicine, Faculty of Health and Medical Sciences, University of Copenhagen, Copenhagen, Denmark; 7 Departments of Psychiatry and Internal Medicine, University Medical Center Groningen, University of Groningen, Groningen, Netherlands

**Keywords:** Functional Somatic Symptoms, Predictors, Adolescent, Infancy physiological regulatory problems, Infancy emotion dysregulation, Infancy contact problems, Maternal postpartum psychiatric illness, Family adversity

## Abstract

Physiological regulatory problems in infancy (i.e., problems with sleeping, feeding, and tactile reactivity) have been associated with impairing Functional Somatic Symptoms (FSS) at ages 5–7. We aimed to extend this finding by examining not only the association of physiological regulatory problems but also other infancy factors (i.e., emotion dysregulation and contact problems) with FSS in pre- and late adolescence. Standardized behavioral assessments and self-report questionnaire data from assessment waves at 0–1, 11–12, and 16–17 years of the population-based Copenhagen Child Cohort (CCC2000) were linked with Danish register data on maternal postpartum psychiatric illness and family adversity as covariates. Multiple linear regression analyses were performed to examine the association between infancy factors and FSS in pre- and late adolescence. Only infancy physiological regulatory problems significantly predicted preadolescent FSS (*b* = 0.38, 95% CI [0.14, 0.62]), also when accounting for maternal postpartum psychiatric illness and family adversity. The association was attenuated for late adolescent FSS.

*Conclusion*: Infancy physiological regulatory problems may represent early signs of a dysregulated stress system and were found to significantly predict FSS in pre- but not late adolescence. Implications for early FSS prevention could include testing interventions promoting infants’ regulation of sleep, feeding, and tactile reactivity.

**Table:** 

**What is Known:** • *Functional Somatic Symptoms (FSS) are common in adolescence.* • *Explanatory models of FSS emphasize a multifactorial etiology involving interactions between early child-related vulnerabilities and contextual factors.* • *Infancy physiological regulatory problems (i.e., problems in the area of sleeping, feeding, and tactile reactivity) might represent early signs of a dysregulated stress system, and have been shown to predict impairing FSS in preschool childhood*.
**What is New:** • *This study investigated the association between infancy factors, namely physiological regulatory problems with feeding, sleeping, and/or tactile reactivity, emotion dysregulation, and contact problems, and FSS in pre- and late adolescence*. • *Only a combination of infancy physiological regulatory problems significantly predicted preadolescent FSS, with the association attenuating for FSS in late adolescence*. • *Implications for early FSS prevention could include parent-mediated interventions aiming to promote infants’ physiological regulatory skills*.

**Supplementary Information:**

The online version contains supplementary material available at 10.1007/s00431-024-05850-7.

## Introduction

Functional Somatic Symptoms, i.e., somatic symptoms that commonly cannot be attributed to a well-defined physical disease, are complex phenomena involving both bodily and brain processes [1]. FSS are common in youth, affecting approximately 25–30% [2, 3]. Symptom presence is similar among boys and girls in early childhood (i.e., at age 5–7), whereas more females tend to report FSS in adolescence [4, 5]. Young children commonly present with only one prominent symptom like stomach pain or headache (i.e., mono-symptomatic), whereas older children and adolescents can also present with several types of symptoms (multi-symptomatic) [6, 7]. Most symptoms are self-limiting, but for around 4–10% of youths in the general population, FSS become burdensome and disabling [2, 3, 8–11].

Current explanatory models for the development of FSS emphasize a multifactorial etiology [1]. This includes child-related vulnerabilities and exposure to an early adverse life context, which are suggested to increase the risk of autonomic dysregulation and alternations in the central processing of sensory input, resulting in bodily distress characterized by FSS [12–15]. Child-related vulnerabilities may include infancy physiological regulatory problems in the areas of sleeping, feeding, and tactile reactivity, potentially representing an immature bodily stress system with disturbed sensory reactivity, which we showed to predict impairing FSS at age 5–7 years in a previous study [14]. Early irregular temperamental style or impaired emotion regulation have also been associated with higher levels of somatic symptoms later [16–24]. In addition, impaired social functioning (i.e., peer socialization difficulties, low social competence) appears to impact the development of somatic symptoms during youth [25–27]. Furthermore, parental history of psychiatric illness and an adverse family environment appear to increase the child’s somatic symptom levels [14, 17, 28–35].

Epidemiological research on the association between early physiological regulatory problems, emotion dysregulation, and contact problems as young as infancy and the development of FSS over time is limited. Understanding these associations could enhance explanatory FSS models, identify at-risk individuals, and guide early intervention efforts. With the current study, we aim to extend the previous findings from the Copenhagen Child Cohort 2000 (CCC2000) on infancy physiological regulatory problems and FSS at age 5–7 years [14], by including additional factors during infancy as predictors of FSS at pre- and late adolescence. Specifically, we examine if physiological regulatory problems, emotion dysregulation, and contact problems in infancy (i.e., age 0–1) will predict FSS at both age 11–12 and age 16–17, also after adjusting for contextual factors like maternal postpartum psychiatric illness and family adversity.

## Materials and methods

### *Study population*

This study was pre-registered on the Open Science Framework platform (Registration DOI: 10.17605/OSF.IO/HB2UE). We used data from the CCC2000 [36], a longitudinal population-based birth cohort study including all children born in one of 16 municipalities in the former Copenhagen County, Denmark (*N* = 6090), which was representative of the Danish child population regarding perinatal and sociodemographic characteristics [36]. The unique Danish civil registration number assigned to all Danish citizens at birth was used to keep track and invite cohort members to participate in the respective follow-up waves. The current study includes data from assessment waves at ages 0–1 years (i.e., infancy), 11–12 (i.e., pre-adolescence), and 16–17 (i.e., late adolescence). Attrition analyses throughout the different assessment waves found lower participation rates in families with lower annual income; immigrant background; single-, smoking-, younger-, and less educated mothers; and a parental- or child psychiatric disorder history [36].

### *Exposure variables*

#### *Infancy factors*

Infancy variables are based on data from community health care nurse records as part of the Danish child health surveillance program, a well-institutionalized component of the Danish healthcare and social welfare system. This program included parental reports and child observation and assessments as part of regular home visits by trained health care nurses throughout the child’s first year of life (*visit 1* at age 1–5 weeks, *visit 2* at age 2–3 months, *visit 3* at age 4–6 months, *visit 4* at age 8–10 months). The individual assessed variables were recorded as either “normal” or “abnormal” by the health nurse. The procedure for child assessments followed a standardized manual to collect information in a systematic manner and to optimize the validity of the assessments [37–39].

#### *Infancy physiological regulatory problems*

In line with the prior CCC2000 study on infancy predictors for impairing FSS at age 5–7 years [14], the variable “infancy physiological regulatory problems” was constructed out of three variables, namely tactile reactivity, feeding, and sleeping. Tactile reactivity was recorded as direct behavioral observations of the child’s reactions by the health care nurses during home visits 1 and 2. Feeding and sleep measures were based on records of direct behavioral observations and parental reports, and were only included from the second month of life (i.e., visits 2, 3, and 4) as most typically developing children first begin to establish a consistent pattern for these behaviors around this age. The constructed final variable had the following categories “no problems” (no feeding, sleeping, or tactile reactivity problems), “one problem” (one problem with either feeding, sleeping, or tactile problems), and “combined problems” (≥ two problems of feeding, sleep, and/or tactile reactivity).

#### *Infancy emotion dysregulation*

The child’s emotion regulation was assessed by the health care nurses at home visits 1–4 [39]. The variable was based on direct observations of the child’s behavior, emotional expressions, and reactions, and registered as normal or abnormal (i.e., abnormal if presenting any deviant pattern of emotions, such as excessive crying or deviant expression of sadness or joy) [40]. The category “abnormal” was considered if indicated by the health care nurse during at least one of the home visits.

#### *Infancy contact problems*

Information on the child’s social contact, communication, and interaction was obtained from the health care nurses records at home visit 4 [39] through the administered standardized BOEL (Blicken Orienterar Efter Ljud) test [41]. We constructed a variable called “contact problems”, which encompassed deviant behavioral patterns in the domains of eye contact, contact smile, and contact babbling, similar to prior CCC2000 research [42]. The category was “abnormal” if indicated as such by the health care nurse in at least one of the assessed domains.

### *Outcome variables*

#### *Functional Somatic Symptoms*

At age 11–12 years, FSS was assessed by the Children’s Somatic Symptoms Inventory (CSSI) [43]. The psychometric properties of the CSSI have been extensively evaluated in various community and clinical samples [43]. In the current sample, internal consistency was good (Cronbach’s *α* = 0.83) [44]. We used the 24-item version where participants were asked “How much were you bothered by (symptom)?” in the last 2 weeks regarding 24 somatic symptom items (e.g., “headaches”), with response options on a 5-point response scale from 0 (“not at all”) to 4 (“a whole lot”). A total sum score of FSS was calculated (score range: 0–96, higher ratings indicating a higher self-reported symptom load).

At age 16–17, the Bodily distress syndrome (BDS) 25-checklist [45] was used to assess FSS. In a former study on the CCC2000 population, we found that the checklist demonstrated high internal consistency (Cronbach’s *α* = 0.90) and satisfactory psychometric quality, representing distinct symptom clusters: cardio-pulmonary, gastro-intestinal, musculoskeletal, and general symptoms [5]. Participants rated 25 somatic symptom items based on how bothered they were by the symptoms in the past year, with responses from 0 (“not at all”) to 4 (“a lot”). The total sum score of FSS ranged from 0 (low) to 100 (high symptom-load).

### *Covariates*

#### *Maternal postpartum psychiatric illness*

Maternal psychiatric illness status in the year after childbirth, i.e., in the years 2000 and 2001, was obtained from the National Psychiatric Central register [46] and categorized as yes/no (“yes” for at least one registered psychiatric diagnosis, comprising of all contacts with psychiatric hospital settings; classified according to the International Classification of Diseases, 10th revision diagnosis of a mental disorder (F00-F99) [47]).

#### *Sex and family adversity*

Sex at birth (categorized as “male”/ “female”) was obtained from the Medical Birth Register [48]. A family adversity index was constructed containing information on: parental place of birth (i.e., both parents born outside Denmark), family composition (i.e., parents did not live together at the time of the child’s birth), maternal age (i.e., a young mother at birth, age ≤ 21), maternal education level (i.e., low education level in the year 2000, i.e., lowest quartile (≤ 25%)), and household income (i.e., a low annual household income in 2000–2001, i.e., lowest quartile (≤ 25%)) with minor adjustments compared to prior work [49]. Variables marked “present/yes” were assigned a score of 1, contributing to a family adversity sum score (range: 0–5, higher scores indicating greater adversity). Due to limited data in higher categories (> 3), we created a binary index: 0 for no family adversity (index score = 0), 1 for any adversity (index scores ≥ 1).

### *Clinical variables*

Information on the presence of a chronic medical condition at age 11–12 years was derived from the parent-reported version of the Soma Assessment Interview (SAI) [50], based on responses to an a priori list of ten well-defined chronic medical conditions (e.g., “diabetes,” “kidney diseases”). Based on the question, “Within the past 12 months, has your child suffered from any of these physical illnesses or handicaps?”, parents were asked whether a physician had diagnosed their child with any of the listed medical conditions, responding with either “yes” or “no.”

### *Statistical analyses*

We performed attrition analyses, comparing participants to non-participants at age 11–12 and 16–17 on infancy, maternal, and psychosocial factors. Subsequently, we performed simple and multiple linear regression analyses to investigate contribution per individual infancy factor and in combination, while increasingly adjusting for contextual factors: Model 1: infancy factors only; Model 2: infancy factors, adding maternal postpartum psychiatric illness as covariate; and Model 3: infancy factors, adding maternal postpartum psychiatric illness and family adversity index as covariates. As an additional exploratory step, we tested if family adversity would interact with the infancy factors to predict FSS. Model assumptions were evaluated by visually inspecting residual- and natural cubic splines plots. Due to violations to the model assumptions, we transformed both FSS at age 11–12 and 16–17 using the square root transformation. As sensitivity analyses, we repeated all multiple linear regression analyses: (1) for each sex separately, (2) excluding all participants with an indication of “yes” to the presence of a chronic medical condition at age 11–12, and (3) only including participants with FSS data at both 11–12 and 16–17. We chose to perform stratified sensitivity analyses instead of adding sex and the presence of a chronic medical condition as further covariates to the statistical models in order to highlight potential differences across groups. In this way, had we observed significant differences in the regression estimates between the main and sensitivity analyses, we would have gained a clearer understanding of the extent to which these factors influence the associations between infancy factors and FSS during adolescence. STATA [51] was used to conduct the analyses. We used an inference level of *α* = 0.05 and employed list-wise deletion to missing data on whole cases.

## Results

### *Sample characteristics*

**Fig. 1:**
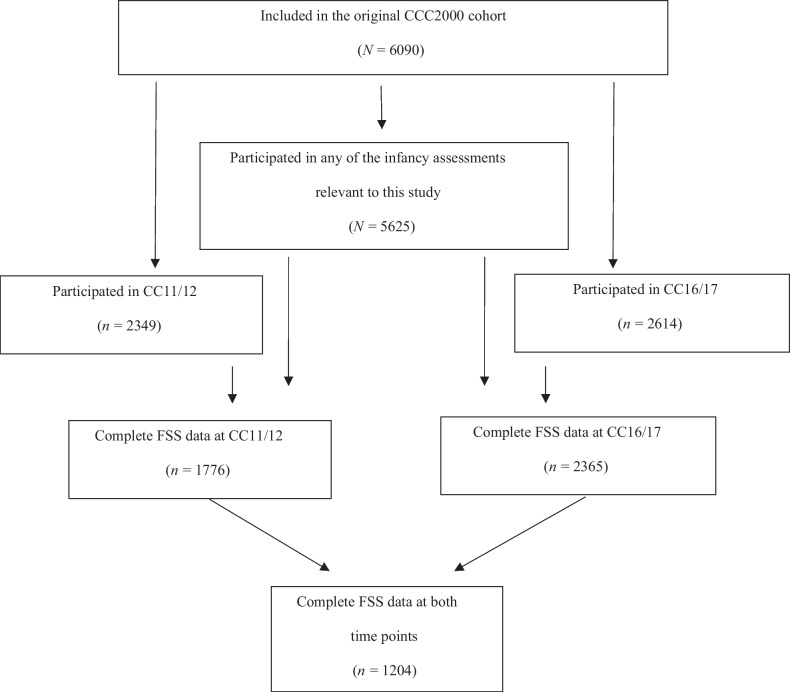
Study flowchart

Figure 1 displays the flow of the current study. The distributions of both age group samples on selected infancy factors, and maternal and psychosocial covariates are summarized in Table 1 as counts and percentages pertaining to individuals participating at the respective outcome age and having data on any of the selected infancy variables. Appendix 1 displays results of attrition analyses at age 11–12 (Table A1) and 16–17 (Table A2) on infancy, maternal, and psychosocial factors. Participants at both age 11–12 and 16–17 had less family adversity during their first year of life compared to non-participants.

**Table 1:** Overview and sample description of infancy, maternal, and psychosocial factors at age 11–12 (*n* = 1776) and 16–17 (*n* = 2365)

Variables	Outcome age	Frequency *n* (%)^a^	Description	Source of information	Distribution *n* (%)
Infancy factors					
Sleeping problems	11–12	1771 (99.7)	Problematic sleeping behavior during at least 1 home visit	Health care nurse records: visit 2, 3, and 4	No problem: 1452 (81.8) Problem: 319 (18.0)
	16–17	2361 (99.8)			No problem: 1948 (82.4) Problem: 413 (17.5)
Feeding problems	11–12	1771 (99.7)	Problematic feeding behavior during at least 1 home visit	Health nurse records: visit 2, 3, and 4	No problem: 1406 (79.2) Problem: 365 (20.6)
	16–17	2355 (99.6)			No problem: 1853 (78.6) Problem: 502 (21.2)
Problems with tactile reactivity	11–12	1694 (95.4)	Problematic behavior while being touched/comforted by parent during at least 1 home visit	Health nurse records: visit 1 and 2	No problem: 1680 (94.6) Problem: 14 (0.8)
	16–17	2247 (95.0)			No problem: 2224 (94.0) Problem: 23 (1.0)
Infancy physiological regulatory problems	11–12	1773 (99.8)	Combined problematic behavior in feeding, sleeping and/or tactile reactivity*	Constructed variable based on the 3 variables above	No problems: 1192 (67.1) 1 problem: 467 (26.3) Combined problems: 114 (6.4)
	16–17	2362 (99.9)			No problems: 1589 (67.2) 1 problem: 617 (26.1) Combined problems: 156 (6.6)
Infancy emotion dysregulation	11–12	1736 (97.8)	Problematic emotional reaction and expression during at least 1 home visit	Health nurse records: visit 1, 2, 3, and 4	Normal: 1518 (85.5) Abnormal: 218 (12.3)
	16–17	2318 (98.0)			Normal: 2040 (86.3) Abnormal: 278 (11.8)
Infancy contact problems	11–12	1776 (100)	Problematic behavior in eye contact, contact smile, and babbling	Health nurse records: visit 4	Normal: 1556 (87.6) Abnormal: 220 (12.4)
	16–17	2365 (100)			Normal: 2064 (87.3) Abnormal: 301 (12.7)
Sex	11–12	1776 (100)	The child’s sex at birth	Medical Birth Register	Male: 844 (47.5) Female: 932 (52.5)
	16–17	2365 (100)			Male: 1043 (44.1) Female: 1322 (55.9)
Presence of a chronic somatic condition	11–12	1645 (92.6)	Indication of “yes” to an a priori list of ten well-defined chronic medical conditions at age 11–12	The Soma Assessment Interview	No: 1434 (87.2) Yes: 211 (12.8)
Maternal-specific factors					
Maternal postpartum psychiatric illness	11–12	1776 (100)	Maternal contact with an in- or outpatient specialized psychiatric facility during the first year after the child’s birth	Psychiatric Central Register	No: 1756 (98.9) Yes: 20 (1.1)
	16–17	2364 (99.9)			No: 2338 (98.9) Yes: 26 (1.1)
Psychosocial factors					
Family adversity	11–12	1768 (99.6)	Constructed variable out of a combination of different socio-demographic adversities	Combination of various Danish national registers	Index score 0: 833 (46.9)
					Index score 1: 935 (52.7)
	16–17	2354 (99.5)			Index score 0: 1057 (44.7)
					Index score 1: 1297 (54.8)

^a^ = The frequency includes individuals participating at the respective outcome age and having data on the specific infancy variable

### *Associations between infancy factors and pre- and late adolescent FSS*

The results of the simple- and multiple linear regression analyses are shown in Tables 2 and 3, respectively. Only combined infancy physiological regulatory problems demonstrated a statistically significant association with FSS at age 11–12 both in the unadjusted (*b* = 0.38, 95% CI [0.14, 0.62]) and in analyses adjusted for maternal-specific and psychosocial covariates (*b* = 0.36 95% CI [0.12, 0.61]). The association was statistically non-significant when considering age 16–17 FSS as outcome in both the unadjusted model (*b* = 0.24, 95% CI [− 0.01, 0.49]) and after adjusting for maternal-specific and psychosocial covariates (*b* = 0.25, 95% CI [0.00, 0.50]). The interaction terms between the infancy factors and the family adversity index as part of the simple linear regression analyses were non-significant for physiological regulatory problems, emotion dysregulation and contact problems at both age 11–12 nor 16–17 (*p*-value range: 0.213–0.807).

**Table 2:** Simple linear regression analyses of infancy, maternal, and psychosocial factors on square root transformed FSS at age 11–12 (*n* = 1776) and 16–17 (*n* = 2365)

Factor	*b*^a^		95% CI		*p*		*n*	
	11*–*12	16–17	11–12	16–17	11–12	16–17	11–12	16–17
Infancy factors								
Infancy physiological regulatory problems							1773; *missing:* 3	2362; *missing:* 3
1 problem	0.11	0.08	− 0.03–0.24	− 0.06–0.22	0.114	0.284		
Combined problems	0.38	0.24	0.14–0.62	− 0.01–0.49	0.002*	0.064		
Infancy emotion (dys)regulation	− 0.05	− 0.08	− 0.23–0.13	− 0.27–0.11	0.576	0.402	1736; *missing: 40*	2318; *missing: 47*
Infancy contact problems	0.003	− 0.01	− 0.15–0.21	− 0.20–0.17	0.742	0.875	1776	2365
Maternal factor								
Maternal postpartum psychiatric illness	0.52	0.01	− 0.04–1.07	− 0.57–0.60	0.067	0.967	1776	2364; *missing: 1*
Psychosocial factors								
Family adversity	0.03	0.05	− 0.10–0.14	− 0.08–0.17	0.671	0.450	1768; *missing: 8*	2354; *missing: 11*

^*a*^Mean difference in square root transformation of FSS, *significant at inference level *α* = 0.05

**Table 3:** Multiple linear regression models of infancy factors on square root transformed FSS at age 11–12 (*n* = 1776) and 16–17 (*n* = 2365), respectively

Model per risk level^b^	Factor	*b*^a^		95% CI		*p*		*n*	
		11–12	16–17	11–12	16–17	11–12	16–17	11–12	16–17
Model 1	Infancy physiological regulatory problems 1 problem	0.11	0.07	–0.03–0.24	–0.07–0.22	0.123	0.300	1736; *Missing: 40*	2318; *Missing: 47*
	Combined problems	0.38	0.24	0.16–0.62	–0.01–0.50	0.002*	0.057		
	Infancy emotion (dys)regulation	–0.08	–0.10	–0.26–0.10	–0.29–0.10	0.380	0.323		
	Infancy contact problems	0.01	–0.02	–0.17–0.20	–0.21–0.16	0.877	0.826		
Model 2	Infancy physiological regulatory problems 1 problem	0.12	0.07	–0.03–0.24	–0.07–0.22	0.120	0.307	1736; *Missing: 40*	2317; *Missing: 48*
	Combined problems	0.38	0.24	0.14–0.62	–0.01–0.50	0.002*	0.056		
	Infancy emotion (dys)regulation	–0.08	–0.10	–0.26–0.19	–0.29–0.10	0.374	0.322		
	Infancy contact problems	0.01	–0.02	–0.17–0.19	–0.21–0.16	0.911	0.827		
	Maternal postpartum psychiatric illness	0.52	0.06	–0.04–1.07	–0.54–0.66	0.067	0.847		
Model 3	Infancy physiological regulatory problems 1 problem	0.11	0.07	–0.03–0.24	–0.07–0.21	0.123	0.327	1728; *Missing: 48*	2307; *Missing: 58*
	Combined problems	0.36	0.25	0.12–0.61	0.00–0.50	0.004*	0.050		
	Infancy emotion (dys)regulation	–0.09	–0.10	–0.27–0.09	–0.29–0.09	0.344	0.313		
	Infancy contact problems	0.01	–0.02	–0.17–0.19	–0.21–0.16	0.909	0.812		
	Maternal postpartum psychiatric illness	0.52	0.07	–0.03–1.08	–0.53–0.66	0.064	0.831		
	Family adversity	0.03	0.05	–0.09–0.15	–0.01–0.17	0.658	0.457		

^a^Mean difference in square root transformed FSS; ^b^Analyses were conducted in a stepwise manner with increasing adjustment as follows: Model 1, infancy factors only; Model 2, infancy factors, adding maternal-specific covariate; and Model 3, infancy factors, adding the maternal-specific and psychosocial covariates; *significant at inference level *α* = 0.05

### *Sensitivity analyses*

Combined infancy physiological regulatory problems showed a positive trend, but not statistically significant association with FSS at age 11–12 in females (*b* = 0.31, 95% CI [− 0.03, 0.65]) (Table A2_1), whereas the result was still statistically significant when including only male participants (*b* = 0.47, 95% CI [0.12, 0.82]). For males only, infancy emotion dysregulation showed a statistically significant, negative association with FSS at age 16–17 (*b* = − 0.29, 95% CI [− 0.57, − 0.01]), also when accounting for maternal postpartum psychiatric illness and family adversity (Table A2_2). Regarding participants without a chronic medical condition at age 11–12, combined infancy physiological regulatory problems (*b* = 0.36, 95% CI [0.09, 0.62]) and maternal postpartum psychiatric illness (*b* = 0.65, 95% CI [0.02, 1.29]) were both statistically significant predictors of preadolescent FSS, also when adjusting for family adversity (see Table A2_3). Lastly, results from additional sensitivity analyses on the sample with FSS data at both 11–12 and 16–17 (*n* = 1204) were comparable to those in the main analyses (see Table A2_4 and A2_5).

## Discussion

### *Main findings*

Within this general population cohort study, combined physiological regulatory problems in infancy significantly predicted FSS at age 11–12 years. This association was attenuated and no longer statistically significant at age 16–17. Emotional dysregulation and contact problems in infancy were not significantly associated with FSS in adolescence. Sex-related differences concerned non-significant findings for all the infancy factors for females. Infancy emotion dysregulation showed a statistically significant, negative, association with late adolescent FSS in males.

### *Interpretation*

Our results of combined infancy physiological regulatory problems as a predictor of preadolescent FSS are in line with our prior finding in relation to preschool FSS using the same CCC2000 cohort [14] and other research reporting an association between early regulatory problems and somatic symptoms in childhood [17, 52, 53]. In adults with conditions characterized by FSS, impairments in bodily systems involved in regulating the stress response, i.e., dysregulation of the autonomic nervous system and the hypothalamic–pituitary axis, have been reported [54]. In younger populations, these physiological aspects could already be at play early in life, expressed as regulation problems and hypersensitivity to sensory stimuli, potentially leading to a continuously activated stress response posing an enduring strain on the body with the development of FSS [14, 15].

The association between combined infancy physiological regulatory problems and FSS was attenuated and no longer statistically significant in late adolescence. Emerging evidence suggests that other psychosocial factors, such as relationship difficulties, neurotic personality tendencies, perceived academic stress, and illness-related worries, may become increasingly salient for the development and manifestation of FSS during late adolescence [9, 55, 56] and might therefore account for the attenuation in our estimates. This idea could also explain the sex-specific differences we observed in this study, as psychological socialization factors and processes are reported to differ for males and females throughout adolescence [57]. Furthermore, the tendency of females to report more somatic symptoms than males is acknowledged already in adolescence [5, 57], and could partly be explained by natural physiological changes occurring during puberty. Here, somatic symptoms appear to increase progressively with pubertal development in females compared to males [12, 58]. Yet, findings of the differential effect of puberty on FSS expression across sexes are contradictory [59]. Furthermore, both combined infancy physiological regulatory problems and maternal postpartum psychiatric illness significantly predicted preadolescent FSS in participants without a chronic medical condition at age 11–12. This finding underscores the potential impact of developing a chronic medical condition later in life, which may mitigate the negative influence associated with having a mother with a psychiatric illness during infancy on subsequent FSS development.

Our non-significant findings concerning impairments in emotion regulation and contact problems with FSS in adolescence in the entire study population and the negative association with FSS at late adolescence in males do not align with the limited previous research [16, 20, 25, 60, 61]. However, this study seems to be the first to investigate these specific impairments as predictors for FSS as early as in infancy, whereas others investigated cross-sectional associations, utilized older participants, or other, yet related, concepts of social contact problems [25–27]. Compared to physiological regulatory problems, these concepts could be more difficult to operationalize and assess reliably during infancy, potentially leading to more attenuated or inconsistent impacts on FSS in this study.

Recommendations for future research include the utilization of more advanced analytical approaches, considering moderation or mediation models, to investigate more complex associations between infancy predictors and contextual factors. Future research could also target to explore infancy physiological regulatory problems and preadolescent FSS more from a developmental perspective, by using a mixed models approach. Unfortunately, the current data does not fully support such an approach, since the CCC2000 study employed a multi-instrument framework to assess concepts of interest such as FSS at various developmental stages (i.e., parent-reports at ages 5–7, self-reports at ages 11–12 and 16–17) to accurately reflect the nuances of growth and change. Lastly, due to the co-occurrence of FSS with internalizing psychopathology [62], it could be fruitful to further investigate these different types of associated symptomatology over time, and how they together are impacted by problematic behaviors already in infancy. This notion is also supported by other lines of research demonstrating an association between early regulation problems and later development of other disorders of behavior and emotion [63], including symptoms of anxiety [64].

### *Strengths and limitations*

We employed data from a large general population-based child cohort, utilizing validated and developmentally appropriate instruments for the assessment of FSS at the different ages, and linked CCC2000 data to Danish national registers. Furthermore, this study included a substantial sample size on all infancy factors.

Limitations include that participants with more family adversity in their first year of life were less likely to participate, potentially hampering the generalizability of findings to less advantaged populations. Furthermore, the psychometric properties of the infancy factors based on health care nurse assessments have not been investigated. While reliability measures are not available, the participation rate was high (94.5%) [14] and the repeated extensive and systematic measures of infant health and data collection as part of the existing child health surveillance program in Denmark represent a unique feature of the current dataset [39]. Nonetheless, defining behavioral and emotional problems in infancy can pose a substantial challenge, and observational coding of behavioral and emotional problems in infancy according to current classification systems warrants further investigation to establish developmental appropriateness for the utility in infancy [65].

In the current study, different measures were used to assess FSS at the different ages (i.e., CSSI at age 11–12, BDS-25 checklist at age 16–17), which could interfere with the generalizability of findings and drawing cohesive conclusions about the impact of infancy regulatory problems on FSS later in life. Register-based information on maternal psychiatric illness only includes diagnoses provided in the hospital setting. Hence, milder forms of maternal psychological problems are not included, as they will typically be managed in primary care settings or not at all.

## Conclusion and implications

This study contributes to limited scientific evidence on associations between infancy factors and the experience of FSS in adolescence. Infancy physiological regulatory problems predicted FSS in preadolescence. Implications for early FSS prevention could include testing parent-mediated interventions promoting infants’ physiological regulatory skills related to sleep, feeding, and tactile reactivity.

## Supplementary Information

Below is the link to the electronic supplementary material.

Supplementary file1 (DOCX 37.5 KB)

## Data Availability

No datasets were generated during the current study.

## Abbreviations

BDS: Bodily distress syndrome
BOEL: Blicken Orienterar Efter Ljud (test)
CCC2000: Copenhagen Child Cohort
CSSI: Children’s Somatic Symptoms Inventory
FSD: Functional Somatic Disorders
FSS: Functional Somatic Symptoms
GDPR: General Data Protection Regulation
SAI: Soma Assessment Interview
