# Correction to: Infancy predictors of functional somatic symptoms in pre- and late adolescence: a longitudinal cohort study

**DOI:** 10.1007/s00431-024-05941-5

**Published:** 2025-01-11

**Authors:** Lina Münker, Martin Køster Rimvall, Lisbeth Frostholm, Eva Ørnbøl, Kaare Bro Wellnitz, Pia Jeppesen, Judith Gerarda Maria Rosmalen, Charlotte Ulrikka Rask

**Affiliations:** 1 Department of Child and Adolescent Psychiatry, Aarhus University Hospital Psychiatry, Palle Juul-Jensens Boulevard 175, 8200 Aarhus N, Denmark; 2 Department of Functional Disorders and Psychosomatics, Aarhus University Hospital, Aarhus, Denmark; 3 Department of Clinical Medicine, Aarhus University, Aarhus, Denmark; 4 Department of Child and Adolescent Psychiatry, Copenhagen University Hospital - Psychiatry Region Zealand, Roskilde, Denmark; 5 Child and Adolescent Mental Health Centre, Copenhagen University Hospital - Mental Health Services CPH, Copenhagen, Denmark; 6 Department of Clinical Medicine, Faculty of Health and Medical Sciences, University of Copenhagen, Copenhagen, Denmark; 7 Departments of Psychiatry and Internal Medicine, University Medical Center Groningen, University of Groningen, Groningen, Netherlands

**Correction to: European Journal of Pediatrics (2024) 184:57**

10.1007/s00431-024-05850-7

We hereby note the following changes made post-publication to the original article, with the title 'Infancy predictors of functional somatic symptoms in pre-and late adolescence: a longitudinal cohort study' (10.1007/s00431-024-05850-7) published in the European Journal of Pediatrics, in numbered order:

*Error*: functional somatic symptom(s)

*Corrected form*: Functional Somatic Symptom(s)

*Error*: Fond in Figure 1 is inconsistent with the fond in the remaining article.

*Corrected form*: The fond has been corrected to be consistent with the fond in the remaining article.

*Error*: The columns in Table 1 (i.e. 'Outcome age', 'Frequency', 'n(%)', 'Description', 'Source of information', 'Distribution n (%)') are left-aligned.

*Corrected form*: These columns in Table 1 are now centralized to increase readability.

*Error*: The columns in Table 2 (i.e. 'b', '95% CI', 'p', 'n') are left-aligned.

*Corrected form*: These columns in Table 2 are now centralized to increase readability.

*Error*: The columns in Table 3 (i.e. 'b', '95% CI', 'p', 'n') are left-aligned.

*Corrected form*: These columns in Table 3 are now centralized to increase readability.

The original article has been corrected.

