# Supplementary material for: Infancy predictors of Functional Somatic Symptoms in pre- and late adolescence: a longitudinal cohort study
Source: Eur J Pediatr. 2024 Dec 2;184(1):57. doi: 10.1007/s00431-024-05850-7 (PMC11611932; doi:10.1007/s00431-024-05850-7)
Supplement: Supplementary file 1 — Supplementary file1 (DOCX 37.5 KB) [file 431_2024_5850_MOESM1_ESM.docx]

Appendices

*Appendix 1: Attrition analyses*

**Table A1**

Attrition analyses of infancy, maternal, and psychosocial factors at age 11-12, comparing participants (*n* = 1776) versus non-participants (*n* = 3849) of those who in any of the infancy assessments relevant to this study (N = 5625).

| Variables | Participants  Frequency *n* (%^a^)  Distribution *n* (%^a^) | Non-participants  Frequency *n* (%^a^)  Distribution *n* (%^a^) | Statistics |
| --- | --- | --- | --- |
| *Infancy factors* |  |  |  |
| Sleeping problems | 1771 (99.7)  1452 (81.8) (No problem)  319 (18.0) (Problem) | 3841 (99.8)  3142 (81.6) (No problem)  699 (18.2) (Problem) | χ2 = 0.03, df(1)  *p* = .867 |
| Feeding problems | 1771 (99.7)  1406 (79.2) (No problem)  365 (20.6) (Problem) | 3831 (99.5)  3006 (78.1) (No problem)  825 (21.4) (Problem) | χ2 = 0.62, df(1)  *p* = .431 |
| Problems with tactile reactivity | 1694 (95.3)  1680 (94.6) (No problem)  14 (0.8) (Problem) | 3709 (96.4)  3667 (95.3) (No problem)  42 (1.1) (Problem) | χ2 = 1.06, df(1)  *p* = .303 |
| Infancy physiological regulatory problems | 1773 (99.8)  1192 (67.1) (No problem)  467 (26.3) (1 problem)  114 (6.4) (Combined problems) | 3844 (99.9)  2573 (66.9) (No problem)  991 (25.8) (1 problem)  280 (7.3) (Combined problems) | χ2 = 1.42, df(2)  *p* = .491 |
| Infancy emotion (dys)regulation | 1736 (97.8)  1518 (85.5) (Normal)  218 (12.3) (Abnormal) | 3744 (97.3)  Normal: 3314 (86.1) (Normal)  430 (11.2) (Abnormal) | χ2 = 1.31, df(1)  *p* = .253 |
| Infancy contact problems | 1776 (100)  1556 (87.6) (Normal)  220 (1.2) (Abnormal) | 3849 (100)  3364 (87.4) (Normal)  485 (12.6) (Abnormal) | χ2 = 0.05, df(1)  *p* = .822 |
| *Maternal-specific factors* |  |  |  |
| Maternal postpartum psychiatric illness | 1776 (100)  1756 (98.9) (No)  20 (1.1) (Yes) | 3848 (99.9)  3793 (98.6) (No)  55 (1.4) (Yes) | χ2 = 0.85, df(1)  *p* = .357 |
| *Psychosocial factors* |  |  |  |
| Family adversity | 1768 (99.6)  833 (46.9) (Index Score 0)  935 (52.7) (Index Score 1) | 3812 (99.0)  1501 (39.0) (Index Score 0)  2311 (60.0) (Index Score 1) | χ2 = 29.74, df(1)  *p* < .001* |

*Note*. ^a^ = relative to group size; * = significant at inference level α = .05

**Table A2**

Attrition analyses of infancy, maternal, and psychosocial factors at age 16-17, comparing participants (*n* = 2365) versus non-participants (*n* = 3260) of those who participated in any of the infancy assessments relevant to this study (N = 5625).

| Variables | Participants  Frequency *n* (%^a^)  Distribution *n* (%^a^) | Non-participants  Frequency *n* (%^a^)  Distribution *n* (%^a^) | Statistics |
| --- | --- | --- | --- |
| *Infancy factors* |  |  |  |
| Sleeping problems | 2361 (99.8)  1948 (82.4) (No problem)  413 (17.5) (Problem) | 3251 (99.7)  2646 (81.2) (No problem)  605 (18.6) (Problem) | χ2 = 1.15, df(1)  *p* = .284 |
| Feeding problems | 2355 (99.6)  1853 (78.4) (No problem)  502 (21.2) (Problem) | 3247 (99.6)  2559 (78.5) (No problem)  688 (21.1) (Problem) | χ2 = 0.01, df(1)  *p* = .908 |
| Problems with tactile reactivity | 2247 (95.0)  2224 (94.0) (No problem)  23 (1.0) (Problem) | 3156 (96.8)  3123 (95.8) (No problem)  33 (1.0) (Problem) | χ2 = 0.01, df(1)  *p* = .0.937 |
| Infancy physiological regulatory problems | 2362 (99.9)  1589 (67.2) (No problem)  617 (26.1) (1 problem)  156 (6.6) (Combined problems) | 3255 (99.9)  2176 (66.8) (No problem)  841 (25.8) (1 problem)  238 (7.3) (Combined problems) | χ2 = 1.06, df(2)  *p* = .590 |
| Infancy emotion (dys)regulation | 2318 (98.0)  2040 (86.3) (Normal)  278 (11.8) (Abnormal) | 3162 (97.0)  2792 (85.6) (Normal)  370 (11.4) (Abnormal) | χ2 = 0.11, df(1)  *p* = .741 |
| Infancy contact problems | 2365 (100)  2064 (87.3) (Normal)  301 (12.7) (Abnormal) | 3260 (100)  2856 (87.6) (Normal)  404 (12.4) (Abnormal) | χ2 = 0.14, df(1)  *p* = .0.708 |
| *Maternal-specific factors* |  |  |  |
| Maternal postpartum psychiatric illness | 2364 (99.9)  2338 (98.9) (No)  26 (1.1) (Yes) | 3260 (100)  3211 (98.5) (No)  49 (1.5) (Yes) | χ2 = 1.69, df(1)  *p* = .193 |
| *Psychosocial factors* |  |  |  |
| Family adversity | 2354 (99.5)  1057 (44.7) (Index Score 0)  1297 (54.8) (Index Score 1) | 3226 (99.0)  1277 (39.2) (Index Score 0)  1949 (59.8) (Index Score 1) | χ2 = 15.82, df(1)  *p* < .001* |

*Note*. ^a^ = relative to group size; * = significant at inference level α = .05

*Appendix 2: Sensitivity analyses*

Table A2_1

Multiple linear regression models of infancy factors on square root transformed FSS at ages 11-12 (n = 1776) and 16-17 (n = 2365), excluding all male participants.

|  |  | b^a^ | | *95% CI* | | *P* | | *N* | |
| --- | --- | --- | --- | --- | --- | --- | --- | --- | --- |
| Model per risk level^b^ | Factor | 11-12 | 16-17 | 11-12 | 16-17 | 11-12 | 16-17 | 11-12 | 16-17 |
| Model 1 | Infancy physiological regulatory problems  1 problem | 0.08 | 0.10 | -0.10 - 0.27 | -0.09 – 0.29 | 0.383 | 0.285 | 910 | 1300 |
|  | Combined problems | 0.31 | 0.33 | -0.03 – 0.65 | -0.02 - 0.67 | 0.071 | 0.062 |  |  |
|  | Infancy emotion (dys)regulation | -0.19 | -0.15 | -0.42 – 0.04 | -0.39 – 0.09 | 0.108 | 0.223 |  |  |
|  | Infancy contact problems | 0.06 | 0.06 | -0.19 – 0.31 | -0.18 – 0.30 | 0.644 | 0.632 |  |  |
| Model 2 | Infancy physiological regulatory problems  1 problem | 0.08 | 0.10 | -0.10 – 0.27 | -0.09 – 0.29 | 0.378 | 0.289 | 910 | 1299 |
|  | Combined problems | 0.32 | 0.32 | -0.02 – 0.65 | -0.02 – 0.67 | 0.065 | 0.064 |  |  |
|  | Infancy emotion (dys)regulation | -0.20 | -0.15 | -0.43 – 0.04 | -0.40 – 0.09 | 0.098 | 0.210 |  |  |
|  | Infancy contact problems | 0.06 | 0.06 | -0.18 – 0.31 | -0.18 – 0.31 | 0.607 | 0.618 |  |  |
|  | Maternal postpartum psychiatric illness | 0.59 | 0.46 | -0.29 – 1.46 | -0.43 – 1.35 | 0.188 | 0.313 |  |  |
| Model 3 | Infancy physiological regulatory problems  1 problem | 0.09 | 0.10 | -0.10 – 0.27 | -0.08 – 0.29 | 0.358 | 0.278 | 906 | 1295 |
|  | Combined problems | 0.30 | 0.31 | -0.04 – 0.64 | -0.03 – 0.66 | 0.088 | 0.072 |  |  |
|  | Infancy emotion (dys)regulation | -0.20 | -0.16 | -0.43 – 0.04 | -0.40 – 0.08 | 0.100 | 0.201 |  |  |
|  | Infancy contact problems | 0.07 | 0.06 | -0.18 – 0.31 | -0.19 – 0.30 | 0.600 | 0.644 |  |  |
|  | Maternal postpartum psychiatric illness | 0.60 | 0.46 | -0.28 – 1.47 | -0.43 – 1.35 | 0.180 | 0.307 |  |  |
|  | Family adversity | 0.08 | 0.15 | -0.09 – 0.24 | -0.01 – 0.32 | 0.350 | 0.071 |  |  |

*Note*. ^a^ = mean difference in square root transformed FSS; ^b^ **=** Analyses were conducted in a stepwise manner with increasing adjustment as follows: Model 1) infancy factors only; Model 2 infancy factors, adding maternal-specific covariate; and Model 3) infancy factors, adding the maternal-specific and psychosocial covariates; * = significant at inference level α = .05

Table A2_2

Multiple linear regression models of infancy factors on square root transformed FSS at ages 11-12 (n = 1776) and 16-17 (n = 2365), excluding all female participants.

|  |  | b^a^ | | *95% CI* | | *P* | | *N* | |
| --- | --- | --- | --- | --- | --- | --- | --- | --- | --- |
| Model per risk level^b^ | Factor | 11-12 | 16-17 | 11-12 | 16-17 | 11-12 | 16-17 | 11-12 | 16-17 |
| Model 1 | Infancy physiological regulatory problems  1 problem | 0.14 | 0.05 | -0.06 - 0.33 | -0.14 – 0.25 | 0.166 | 0.573 | 826 | 1018 |
|  | Combined problems | 0.47 | 0.28 | 0.12 – 0.82 | -0.04 - 0.61 | 0.008* | 0.089 |  |  |
|  | Infancy emotion (dys)regulation | 0.00 | -0.29 | -0.28 – 0.29 | -0.57 – -0.01 | 0.984 | 0.044* |  |  |
|  | Infancy contact problems | -0.03 | -0.14 | -0.29 – 0.23 | -0.39 – 0.11 | 0.815 | 0.261 |  |  |
| Model 2 | Infancy physiological regulatory problems  1 problem | 0.14 | 0.05 | -0.06 – 0.33 | -0.14 – 0.25 | 0.163 | 0.573 | 826 | 1018 |
|  | Combined problems | 0.47 | 0.28 | 0.12 – 0.82 | -0.04 – 0.61 | 0.008* | 0.089 |  |  |
|  | Infancy emotion (dys)regulation | 0.00 | -0.29 | -0.28 – 0.29 | -0.57 – -0.01 | 0.962 | 0.044* |  |  |
|  | Infancy contact problems | -0.05 | -0.14 | -0.30 – 0.21 | -0.39 – 0.11 | 0.725 | 0.262 |  |  |
|  | Maternal postpartum psychiatric illness | 0.56 | 0.00 | -0.15 – 1.27 | -0.71 – 0.72 | 0.123 | 0.995 |  |  |
| Model 3 | Infancy physiological regulatory problems  1 problem | 0.13 | 0.05 | -0.06 – 0.33 | -0.14 – 0.24 | 0.176 | 0.606 | 822 | 1012 |
|  | Combined problems | 0.45 | 0.29 | 0.10 – 0.80 | -0.04 – 0.62 | 0.012* | 0.083 |  |  |
|  | Infancy emotion (dys)regulation | -0.00 | -0.29 | -0.29 – 0.28 | -0.57 – -0.00 | 0.976 | 0.048* |  |  |
|  | Infancy contact problems | -0.05 | -0.15 | -0.31 – 0.21 | -0.40 – 0.11 | 0.718 | 0.253 |  |  |
|  | Maternal postpartum psychiatric illness | 0.56 | -0.01 | -0.15 – 1.27 | -0.73 – 0.70 | 0.124 | 0.970 |  |  |
|  | Family adversity | -0.00 | -0.07 | -0.17 – 0.17 | -0.23 – 0.10 | 0.976 | 0.442 |  |  |

*Note*. ^a^ = mean difference in square root transformed FSS; ^b^ **=** Analyses were conducted in a stepwise manner with increasing adjustment as follows: Model 1) infancy factors only; Model 2 infancy factors, adding maternal-specific covariate; and Model 3) infancy factors, adding the maternal-specific and psychosocial covariates; * = significant at inference level α = .05

Table A2_3

Multiple linear regression models of infancy factors on square root transformed FSS at ages 11-12 (n = 1776) and 16-17 (n = 2365), excluding all participants with a chronic somatic condition at age 11-12.

|  |  | b^a^ | | *95% CI* | | *P* | | *N* | |
| --- | --- | --- | --- | --- | --- | --- | --- | --- | --- |
| Model per risk level^b^ | Factor | 11-12 | 16-17 | 11-12 | 16-17 | 11-12 | 16-17 | 11-12 | 16-17 |
| Model 1 | Infancy physiological regulatory problems  1 problem | 0.04 | 0.16 | -0.10 - 0.19 | -0.05 – 0.36 | 0.563 | 0.137 | 1401;  *Missing*: 375 | 1037;  *Missing*: 1328 |
|  | Combined problems | 0.36 | 0.10 | 0.09 – 0.62 | -0.27 - 0.48 | 0.008* | 0.585 |  |  |
|  | Infancy emotion (dys)regulation | -0.07 | -0.20 | -0.27 – 0.13 | -0.48 – 0.09 | 0.500 | 0.176 |  |  |
|  | Infancy contact problems | 0.07 | -0.06 | -0.27 – 0.12 | -0.32 – 0.21 | 0.456 | 0.683 |  |  |
| Model 2 | Infancy physiological regulatory problems  1 problem | 0.05 | 0.16 | -0.10 – 0.19 | -0.05 – 0.36 | 0.547 | 0.137 | 1401;  *Missing*: 375 | 1037;  *Missing*: 1328 |
|  | Combined problems | 0.36 | 0.10 | 0.09 – 0.62 | -0.27 – 0.48 | 0.008* | 0.595 |  |  |
|  | Infancy emotion (dys)regulation | -0.06 | -0.20 | -0.26 – 0.13 | -0.48 – 0.09 | 0.527 | 0.175 |  |  |
|  | Infancy contact problems | -0.08 | -0.05 | -0.28 – 0.11 | -0.32 – 0.21 | 0.397 | 0.689 |  |  |
|  | Maternal postpartum psychiatric illness | 0.65 | -0.22 | 0.02 – 1.29 | -1.09 – 0.64 | 0.045* | 0.616 |  |  |
| Model 3 | Infancy physiological regulatory problems  1 problem | 0.04 | 0.16 | -0.10 – 0.19 | -0.05 – 0.37 | 0.553 | 0.133 | 1394;  *Missing*: 382 | 1033;  *Missing*: 1332 |
|  | Combined problems | 0.33 | 0.12 | 0.06 – 0.60 | -0.26 – 0.50 | 0.015* | 0.542 |  |  |
|  | Infancy emotion (dys)regulation | -0.07 | -0.18 | -0.27 – 0.13 | -0.47 – 0.10 | 0.474 | 0.206 |  |  |
|  | Infancy contact problems | -0.08 | -0.06 | -0.27 – 0.11 | -0.32 – 0.21 | 0.398 | 0.669 |  |  |
|  | Maternal postpartum psychiatric illness | 0.68 | -0.21 | 0.04 – 1.32 | -1.07 – 0.66 | 0.038* | 0.640 |  |  |
|  | Family adversity | 0.07 | 0.05 | -0.06 – 0.20 | -0.13 – 0.23 | 0.300 | 0.569 |  |  |

*Note*. ^a^ = mean difference in square root transformed FSS; ^b^ **=** Analyses were conducted in a stepwise manner with increasing adjustment as follows: Model 1) infancy factors only; Model 2 infancy factors, adding maternal-specific covariate; and Model 3) infancy factors, adding the maternal-specific and psychosocial covariates; * = significant at inference level α = .05

**Table A2_4**

Simple linear regression analyses of infancy, maternal, and psychosocial factors on square root transformed FSS at age 11-12 and 16-17 (*n* = 1204, participants with complete FSS data at both age points, given that they participated in any infancy assessments)

|  | b^a^ | | *95% CI* | | *P* | | *N* | |
| --- | --- | --- | --- | --- | --- | --- | --- | --- |
| Factor | 11-12 | 16-17 | 11-12 | 16-17 | 11-12 | 16-17 | 11-12 | 16-17 |
| *Infancy factors*  Infancy physiological regulatory problems  1 problem | 0.08 | 0.07 | -0.09 – 0.24 | -0.12 – 0.27 | 0.351 | 0.464 | 1202; *Missing:* 2 | |
| Combined problems | 0.34 | 0.04 | 0.04 – 0.64 | -0.32 – 0.39 | 0.026* | 0.838 |  |  |
| Infancy emotion (dys)regulation | -0.03 | -0.09 | -0.25 – 0.19 | -0.35 – 0.16 | 0.806 | 0.472 | 1178; *Missing: 26* | |
| Infancy contact problems | 0.03 | -0.11 | -0.18 – 0.25 | -0.36 – 0.14 | 0.754 | 0.393 | 1204 | |
| *Maternal factor* |  |  |  |  |  |  |  |  |
| Maternal postpartum psychiatric illness | 0.29 | -0.06 | -0.40 – 0.97 | -0.87 – 0.75 | 0.409 | 0.880 | 1204 | |
| *Psychosocial factors* |  |  |  |  |  |  |  |  |
| Family adversity | 0.08 | -0.02 | -0.06 – 0.22 | -0.19 – 0.15 | 0.269 | 0.825 | 1199; *Missing: 5* | |

*Note*. *b^a^* = mean difference in square root transformation of FSS, * = significant at inference level α = .05

Table A2_5

Multiple linear regression models of infancy, maternal, and psychosocial factors on square root transformed FSS at age 11-12 and 16-17 (n = 1204, participants with complete FSS data at both age points, given that they participated in any infancy assessments).

|  |  | b^a^ | | *95% CI* | | *P* | | *N* | |
| --- | --- | --- | --- | --- | --- | --- | --- | --- | --- |
| Model per risk level^b^ | Factor | 11-12 | 16-17 | 11-12 | 16-17 | 11-12 | 16-17 | 11-12 | 16-17 |
| Model 1 | Infancy physiological regulatory problems  1 problem | 0.07 | 0.08 | -0.10 - 0.24 | -0.12 – 0.27 | 0.378 | 0.447 | 1178; *Missing: 26* | |
|  | Combined problems | 0.34 | 0.05 | -0.04 – 0.64 | -0.30 - 0.41 | 0.028* | 0.764 |  |  |
|  | Infancy emotion (dys)regulation | -0.04 | -0.09 | -0.27 – 0.18 | -0.35 – 0.17 | 0.693 | 0.515 |  |  |
|  | Infancy contact problems | 0.01 | -0.11 | -0.20 – 0.23 | -0.36 – 0.15 | 0.909 | 0.411 |  |  |
| Model 2 | Infancy physiological regulatory problems  1 problem | 0.07 | 0.08 | -0.10 – 0.27 | -0.12 – 0.27 | 0.380 | 0.446 | 1178; *Missing: 26* | |
|  | Combined problems | 0.34 | 0.05 | -0.02 – 0.65 | -0.30 – 0.41 | 0.027* | 0.767 |  |  |
|  | Infancy emotion (dys)regulation | -0.05 | -0.09 | -0.43 – 0.04 | -0.35 – 0.17 | 0.686 | 0.516 |  |  |
|  | Infancy contact problems | 0.01 | -0.11 | -0.20 – 0.23 | -0.36 – 0.15 | 0.914 | 0.412 |  |  |
|  | Maternal postpartum psychiatric illness | 0.30 | -0.06 | -0.39 – 0.99 | -0.87 – 0.75 | 0.389 | 0.886 |  |  |
| Model 3 | Infancy physiological regulatory problems  1 problem | 0.07 | 0.08 | -0.12 – 0.27 | -0.09 – 0.24 | 0.398 | 0.435 | 1173; *Missing:31* | |
|  | Combined problems | 0.32 | 0.07 | -0.29 – 0.43 | 0.02 – 0.62 | 0.040* | 0.693 |  |  |
|  | Infancy emotion (dys)regulation | -0.05 | -0.08 | -0.34 – 0.18 | -0.27 – 0.18 | 0.676 | 0.563 |  |  |
|  | Infancy contact problems | 0.01 | -0.11 | -0.36 – 0.15 | -0.21 – 0.23 | 0.923 | 0.409 |  |  |
|  | Maternal postpartum psychiatric illness | 0.32 | -0.06 | -0.87 – 0.75 | -0.37 – 1.01 | 0.365 | 0.885 |  |  |
|  | Family adversity | 0.08 | -0.01 | -0.18 – 0.16 | -0.07 – 0.22 | 0.292 | 0.930 |  |  |

*Note*. ^a^ = mean difference in square root transformed FSS; ^b^ **=** Analyses were conducted in a stepwise manner with increasing adjustment as follows: Model 1) infancy factors only; Model 2 infancy factors, adding maternal-specific covariate; and Model 3) infancy factors, adding the maternal-specific and psychosocial covariates; * = significant at inference level α = .05
